# Beyond cartilage degeneration: osteoarthritis as a systems failure of inflammation regulation

**DOI:** 10.3389/fmed.2026.1843245

**Published:** 2026-05-28

**Authors:** David Bar-Or, Jason Williams, Melissa Hausburg, Greg Thomas, Raphael Bar-Or

**Affiliations:** Department of Trauma Research, Swedish Medical Center, Englewood, CO, United States

**Keywords:** aging, COX-2, inflammation, MRP4, osteoarthritis, PGE2, PP2A

## Abstract

Osteoarthritis (OA) is traditionally framed as a degenerative cartilage disease caused by age-related mechanical wear. However, this paradigm does not adequately account for the synovitis, metabolic disturbance, and pain that commonly precede overt structural loss. In this Hypothesis and Theory article, we propose a testable systems model in which OA reflects a failure of interdependent synovial regulatory networks rather than a purely structural endpoint. These networks include: (1) energy-redox imbalance driven by declining NAD+ availability and mitochondrial inefficiency, which may prime synovial inflammation; (2) dysregulated MRP4- and MCT4-dependent efflux, leading to extracellular accumulation of prostaglandins and lactate that may promote nociception and stromal activation; (3) reduced protein phosphatase 2A (PP2A)-mediated signaling restraint, which may permit persistence of NF-κB and MAPK phosphorylation; and (4) impaired lipid mediator class switching, which may limit biosynthesis of specialized pro-resolving mediators such as Maresin-2 and Resolvin D1. We further propose that microtubule organization may function as a mechanometabolic interface linking redox decline, lactate signaling, transporter localization, and impaired mechanosensing. Because current anti-inflammatory therapies largely suppress inflammatory output rather than restore regulatory control, they may provide symptomatic relief without durable disease modification. We therefore outline a regulatory-restoration framework focused on NAD+ -redox alignment, mediator transport, phosphatase tone, and resolution-pathway recovery, while explicitly defining the limitations and falsifiable predictions of the model.

## 1 Introduction: reframing OA

Defining osteoarthritis (OA) as a degenerative joint disease oversimplifies the complex interplay of physical, biochemical, metabolic, and inflammatory forces that drive cartilage breakdown and structural failure. Mechanical loading remains central to OA biology, but mechanical stress becomes more pathogenic when intrinsic mechanisms of inflammatory control and repair fail. OA can occur in joints in which mechanical loading is not the only explanatory variable (1). Contemporary research has therefore sought to reconcile structural degeneration with synovitis and pain by framing OA as a low-grade inflammatory disease (2), leading to therapeutic strategies centered on cytokine suppression or cyclooxygenase-2 (COX-2) inhibition (3). However, targeting these downstream mediators often fails to restore resolution, and disease progression can continue because the regulatory mechanisms governing inflammatory termination remain incompletely addressed.

Because this article is a Hypothesis and Theory contribution, the proposed model is intended to organize existing evidence and generate testable predictions rather than to claim that all four axes have already been causally validated in OA. Throughout the manuscript, we distinguish direct OA-specific evidence from indirect support derived from aging biology, inflammation biology, and related disease systems.

Rather than isolating a single mechanical or inflammatory mechanism, we propose reframing OA as a disorder of regulatory failure. This model integrates multiple interconnected systems that degrade secondary to aging and injury. Under normal conditions, joint use generates low-level mechanical stress and damage-associated molecular patterns (DAMPs) in a process of sterile inflammation (4). In healthy joints, this sterile inflammation is successfully resolved to maintain immune homeostasis. The clinical challenge arises when the return to homeostasis is impaired by aging or stressors such as obesity, overuse, and injury. Indeed, joint injury early in life significantly increases the risk of early-onset OA and elevates the lifetime risk of the condition (5). Consequently, OA prevalence scales with age, with the primary risk factors identified as obesity, metabolic disease, and aging (6).

We suggest a mechanistic framework for OA grounded in age-related biochemical decline and metabolic dysfunction, characterized by persistent inflammation and impaired resolution. While chondrocytes remain central to joint homeostasis through the synthesis and maintenance of articular cartilage, we expand this perspective to encompass the broader joint environment—including synoviocytes, macrophages, and the synovial fluid. Each of these components is critically impacted by metabolic failure, energy–redox imbalance, and a progressive loss of the innate capacity to resolve inflammation.

## 2 Aging and redox dysregulation as the origin of inflammatory dysregulation in osteoarthritis

Aging is the primary risk factor for OA, yet the biological mechanisms underlying this susceptibility remain incompletely defined (7). While OA has historically been attributed to inflammatory cytokines such as IL-1β and TNF-α (8), the limited success of clinical trials targeting these specific mediators suggests that joint inflammation is driven by broader regulatory failures (2, 9, 10). The traditional wear-and-tear perspective is increasingly replaced by an understanding of the profound metabolic and molecular alterations inherent to the aging joint (7, 11), suggesting that age-associated metabolic and redox instability may be upstream contributors to joint vulnerability.

A hallmark of aging is “inflammaging”—a chronic, low-grade sterile inflammation (12) fueled by progressive metabolic instability. Intracellular levels of NAD+ decline with age due to heightened consumption by CD38, sirtuins, and PARP-mediated DNA repair, alongside diminished salvage capacity (13, 14). Replenishing NAD+ can reverse aspects of this dysfunction, primarily through sirtuin-dependent homeostatic pathways (15, 16). This decline is compounded by age-related mitochondrial inefficiency, characterized by reduced oxidative phosphorylation and altered reactive oxygen species (ROS) production (17, 18). Because mitochondrial metabolism is the primary route for NADH oxidation, its impairment disrupts the ratio, forcing a compensatory shift toward glycolysis to maintain levels (19, 20). This “Warburg-like” state results in a pseudo-hypoxic microenvironment lactate accumulation (16).

These disturbances are evident in OA tissues; chondrocytes and synovial fibroblasts exhibit impaired respiratory chain activity, altered membrane potential, and increased oxidative stress compared with healthy controls (21–23). This metabolic reprogramming toward glycolysis is a hallmark of the OA joint environment (24). Notably, mechanical injury induces nearly identical mitochondrial and oxidative failures, suggesting that post-traumatic and age-related OA may converge on a shared metabolic pathology (25–27).

Lactate should not be framed solely as a harmful waste product. It is a signaling metabolite with context-dependent functions. Increased glycolysis drives lactate export via MCT4, acidifying the synovial microenvironment and influencing macrophage polarization (28, 29). Beyond extracellular effects, lactate can influence gene expression through lysine lactylation (30). Histone lactylation in macrophages can promote pro-resolving M2 phenotypes (30, 31), but lactylation more broadly reflects metabolic-epigenetic reprogramming that can support diverse transcriptional outputs depending on cellular context (31, 32). In the glycolytic OA joint, lactylation may therefore represent either a compensatory response or a maladaptive persistence signal, a distinction requiring direct experimental testing.

## 3 Loss of protein phosphatase 2A disrupts kinase restraint and perpetuates chronic inflammation in OA

We propose that reduced protein phosphatase 2A (PP2A) activity represents a candidate regulatory node that may contribute to persistent inflammatory signaling and catabolic progression in OA. In healthy tissue, PP2A functions as a physiological brake on stress-responsive kinase pathways, including NF-κB and MAPK signaling (33, 34). We hypothesize that oxidative stress and metabolic dysfunction in the OA microenvironment may impair PP2A activity through post-translational modifications and disruption of holoenzyme assembly. This loss of phosphatase tone could produce phosphorylation drift, a state of sustained phosphorylation that favors inflammatory and catabolic gene expression even after the initiating stimulus has waned.

PP2A is a ubiquitous serine/threonine phosphatase organized as a heterotrimeric complex comprising a scaffolding A subunit, a catalytic C subunit, and a regulatory B subunit (35, 36). The diversity of B subunit classes dictates subcellular localization and substrate specificity (35, 37, 38). In healthy tissues, kinase activation is transient; PP2A-mediated dephosphorylation restores signaling toward a resting state, preventing persistence of acute-phase responses (39). Key targets include ERK1/2, AKT, p38 MAPK, and NF-κB components such as IKKβ and RelA (40, 41).

We also acknowledge that PP2A is unlikely to act in isolation. Other regulatory mechanisms, including persistent kinase activation, additional phosphatases, altered mechanotransduction, epigenetic reprogramming, cellular senescence, innate immune activation, and mitochondrial dysfunction, may interact with or operate independently of PP2A. The proposed role of PP2A should therefore be interpreted as one candidate node within a larger regulatory network rather than as a proven master regulator of OA.

We propose that two primary regulatory layers of PP2A may be compromised in the OA joint. First, reversible carboxyl methylation of the catalytic C subunit at Leu309, catalyzed by LCMT1 and reversed by PME-1, is essential for recruiting B-type subunits and determining substrate targeting (42, 43). Metabolic stress and redox imbalance may disrupt one-carbon metabolism and S-adenosylmethionine (SAM) availability, potentially shifting holoenzyme assembly toward less specific or less active states. Second, PP2A is sensitive to oxidative conditions. Evidence from neuronal and other systems indicates that the catalytic subunit contains vicinal thiol groups susceptible to disulfide bond formation, tyrosine nitration, and S-glutathionylation, all of which can inhibit phosphatase function (44–46). Direct measurement of these modifications in OA synoviocytes and chondrocytes remains limited and is therefore a central test of this hypothesis. The accumulation of advanced glycation end-products and lipid peroxidation products in aging synovium may further inhibit PP2A through covalent modification (47–49).

Functional impairment of PP2A may amplify pathological circuits within the joint. Persistent phosphorylation of NF-κB p65 and MAPKs maintains expression of matrix metalloproteinases such as MMP-13, accelerating type II collagen degradation and proteoglycan loss (50, 51). This catabolic activity creates a feed-forward loop, as extracellular matrix breakdown products further activate pattern recognition receptors. Sustained AKT and MAPK signaling may also drive chondrocyte phenotypic drift, pushing cells toward hypertrophic differentiation and senescence-associated inflammatory signaling (52–54). However, the direction and magnitude of these effects remain context-dependent and require direct OA-specific validation.

Direct measurements of PP2A activity in OA joint tissue are sparse; however, this hypothesis is supported by convergent evidence: overactivation of known PP2A-constrained pathways, presence of inhibitory conditions such as ROS and advanced glycation end-products in the OA joint, and preliminary evidence that PP2A modulates apoptosis and TGF-β1 signaling in human chondrocytes (55, 56). Restoring phosphatase tone through small-molecule activators or inhibition of endogenous PP2A antagonists remains a conceptual therapeutic strategy rather than an established OA treatment (38). Future OA-specific studies should quantify PP2A activity, methylation status, oxidative modification, regulatory B-subunit composition, and downstream kinase phosphorylation in matched synovium, cartilage, and synovial fluid samples.

## 4 Transport bias as a driver of inflammatory persistence

If redox imbalance establishes inflammatory readiness in OA, transport dysregulation may determine the spatial persistence of inflammatory signaling. Cellular transport systems are not passive conveyors of metabolites; they actively shape the extracellular signaling environment, producing spatially organized field effects that can drive synovitis and pain. We define transport bias as an imbalance between mediator export, reuptake, and intracellular retention that shifts inflammatory mediators such as PGE2 and lactate toward persistent extracellular signaling.

Current OA pharmacology attempts to control prostanoid output through COX-2 inhibition, yet these strategies do not produce consistent disease modification (57). PGE2, once synthesized, can be exported via the ATP-binding cassette transporter MRP4 (ABCC4) to activate EP receptors and propagate inflammation (58, 59). We hypothesize that in inflamed OA synoviocytes, increased MRP4-mediated export and impaired PGT-mediated reuptake could create asymmetric extracellular PGE2 accumulation. This proposed transport-driven gradient is testable by measuring MRP4 expression, plasma membrane localization, PGE2 efflux, PGT activity, and intracellular-to-extracellular PGE2 ratios in primary OA synoviocytes.

Lactate follows similar regulatory logic. Although often interpreted as a biomarker of hypoxia or mitochondrial failure (60), lactate is a signaling metabolite exported through MCT4. Extracellular lactate can promote angiogenesis, enhance nociceptor activity, and influence macrophage polarization (61, 62). Consequently, chronic synovitis may reflect persistent extracellular gradients sustained by transporter activity rather than simple overproduction (63).

This transport bias is governed at two coupled levels: gene expression of transporters and microtubule-dependent trafficking that sets transporter abundance at the plasma membrane. Microtubules serve as a cellular execution layer that converts metabolic stress into signaling behavior, because long-lived, acetylated microtubules serve as preferred tracks for motor-driven cargo delivery (64, 65). In the OA synovium, inflammatory signaling may therefore include a spatial logistics problem: receptors and transporters must be delivered and recycled at specific membrane domains. Central to this process is HDAC6, the dominant cytosolic alpha-tubulin deacetylase that links inflammatory tone to the microtubule state (66).

The post-translational modification repertoire of alpha-tubulin has expanded to include alpha-tubulin lactylation, which can be catalyzed by HDAC6 (67). In OA, this suggests that lactate may not only reprogram chromatin but may also affect intracellular transport capacity through tubulin modifications. This remains a mechanistic hypothesis rather than an established OA mechanism and should be tested directly by assessing tubulin acetylation and lactylation, HDAC6 activity, transporter localization, and PGE2/lactate efflux in OA-relevant cells.

## 5 Failure of resolution as the defining feature of chronic osteoarthritis

If redox imbalance primes inflammation and transport bias amplifies it, neither mechanism alone explains why OA fails to terminate inflammation over time. Healthy inflammation is an active, self-limiting program that initiates, amplifies, and then resolves through a biochemically programmed sequence driven by specialized pro-resolving mediators (SPMs), including Maresins, Resolvins, and Protectins (68, 69). Failure of resolution refers to impaired activation of endogenous biochemical pathways that normally terminate inflammation and restore tissue homeostasis; it is not equivalent to passive decay of inflammatory signaling.

In healthy inflammatory responses, lipid mediator class switching coordinates the transition from prostaglandin- and leukotriene-dominant initiation toward SPM generation. COX-2, 5-LOX, 12-LOX, and 15-LOX contribute to this switch by converting arachidonic acid and docosahexaenoic acid into mediators that first amplify and then resolve inflammation (70–72). Efferocytosis, the phagocytic clearance of apoptotic inflammatory cells, further supports resolution by inducing pro-resolving lipid mediator biosynthesis (73–76).

In OA, lipid mediator class switching appears stunted. Low synovial fluid levels of pro-resolving mediators have been associated with disease severity and pain (77). We hypothesize that this resolution failure may function as a downstream integration point rather than a stand-alone mechanism: excessive prostaglandin efflux may prevent intracellular signals needed to induce 12/15-LOX expression; PP2A suppression may sustain inflammatory transcription and inhibit pro-resolving gene programs; and redox-mitochondrial stress may compromise biosynthetic capacity.

This failure of resolution explains why inflammation in OA can persist in the absence of infection or classic autoimmunity. Chronic inflammation may reflect impaired termination rather than excessive activation. NSAIDs and COX-2 inhibitors block prostaglandin production, but in some contexts, prostaglandins also participate in the transition toward resolution (78, 79). Similarly, glucocorticoids suppress transcriptional amplification but do not necessarily restore resolution machinery. Consequently, OA may persist not because inflammation is too strong in a simple linear sense, but because the regulatory mechanisms required to terminate and repair inflammatory responses are insufficient.

## 6 A systems model of osteoarthritis pathogenesis and future directions

The four domains are best viewed as interacting control failures rather than a mandatory temporal sequence. Redox instability establishes susceptibility; transport bias localizes and sustains extracellular mediator gradients; phosphorylation drift maintains transcriptional activation; and resolution failure prevents termination of the response. These events may culminate in synovitis, cartilage degradation, fibrosis, subchondral remodeling, and pain as convergent consequences of shared regulatory collapse rather than isolated disease processes.

This framework clarifies why synovitis can predict progression more accurately than cartilage damage, why OA persists without traditional autoimmunity, and why risk scales steeply with age. It also explains the limited disease-modifying efficacy of conventional anti-inflammatory strategies such as COX-2 inhibition and cytokine blockade. These approaches may provide transient symptom relief, but suppressing inflammatory output does not necessarily rebuild the joint’s intrinsic capacity to regulate and resolve inflammation.

Accordingly, future disease modification should prioritize control restoration across four therapeutic axes: re-establishing redox homeostasis through NAD+ -redox support and mitochondrial stabilization; normalizing mediator transport to collapse pathological PGE2 and lactate gradients while preserving intracellular lipid dynamics; restoring phosphatase tone through PP2A reactivation or removal of endogenous PP2A inhibition; and enabling resolution by recovering 12/15-LOX function and promoting mediator class switching. These strategies remain preclinical hypotheses and should not be interpreted as clinically actionable recommendations until validated in OA-specific models and clinical studies.

### 6.1 Testable predictions

*Prediction 1*: OA synoviocytes and chondrocytes should show measurable NAD+/NADH imbalance, mitochondrial inefficiency, increased glycolytic flux, and lactate export compared with non-OA or less inflamed controls.

*Prediction 2*: PP2A activity should be reduced in OA-relevant cells and should correlate with oxidative modifications, altered methylation state, or disrupted holoenzyme composition.

*Prediction 3*: Inflamed OA synoviocytes should demonstrate transporter bias, including altered MRP4/MCT4 abundance, plasma membrane localization, and extracellular accumulation of PGE2 and lactate.

*Prediction 4*: Microtubule post-translational modifications, including alpha-tubulin acetylation and potentially lactylation, should correlate with transporter localization and inflammatory persistence.

*Prediction 5*: Synovial fluid or tissue signatures should show reduced SPM abundance or impaired 12/15-LOX-dependent resolution capacity, especially in patients with active synovitis and pain (see Table 1).

**Table 1:** Evidence hierarchy and experimental validation strategy for the proposed OA regulatory-failure model.

Regulatory axis	OA-specific support	Extrapolated/indirect support	Proposed validation
Energy-redox imbalance	OA chondrocytes and synovial fibroblasts show mitochondrial dysfunction, oxidative stress, and glycolytic reprogramming.	Aging-related NAD+ decline, pseudo-hypoxia, and inflammaging provide broader biological context.	Measure NAD+/NADH, respiration, ROS, glycolytic flux, and lactate export in matched OA tissues.
Transport bias	PGE2 and lactate are relevant to OA synovitis, pain, and stromal activation.	MRP4-mediated prostaglandin efflux and MCT4-mediated lactate export are established in other inflammatory/metabolic systems.	Quantify MRP4, PGT, MCT4, membrane localization, intracellular/extracellular PGE2, and lactate gradients.
PP2A/phosphorylation drift	OA tissues show activation of PP2A-constrained pathways such as NF-κB and MAPK.	PP2A regulates inflammatory signaling in immune, neuronal, and cancer systems.	Measure PP2A activity, methylation, oxidative modification, B-subunit composition, and downstream phosphorylation.
Resolution failure	Synovial-fluid SPM abundance and lipid mediator balance may relate to pain and inflammatory activity.	SPM biology and efferocytosis are well described in inflammatory resolution.	Profile 12/15-LOX activity, SPMs, efferocytosis markers, and response to resolution-directed interventions.

### 6.2 Boundaries and limitations of the hypothesis

Several limitations should be emphasized. First, the model is not intended to exclude mechanical loading, malalignment, trauma, genetics, or obesity; rather, it proposes that these inputs become disease-sustaining when regulatory recovery fails. Second, several mechanisms, particularly PP2A suppression, MRP4/PGT imbalance, and tubulin lactylation in OA, require direct experimental validation in human OA tissues. Third, the model may not apply equally to all OA phenotypes; metabolic OA, post-traumatic OA, erosive hand OA, and advanced end-stage OA may differ in the relative contribution of each axis. Fourth, therapeutic restoration of these pathways must avoid impairing necessary host defense or repair responses. These limitations define the experimental agenda rather than weaken the hypothesis.

### 6.3 Biomarker and translational strategy

A regulatory model requires biomarkers of system capacity rather than inflammatory output alone. Future diagnostics should emphasize NAD+/NADH balance, mitochondrial respiration, PP2A activity, transporter localization and export indices, PGE2 intracellular-to-extracellular ratios, lactate gradients, and synovial-fluid SPM abundance. Tracking these measures may enable mechanism-aligned patient stratification and precision testing of therapies designed to rebuild the joint’s control architecture and achieve durable resolution.

**Figure 1:**
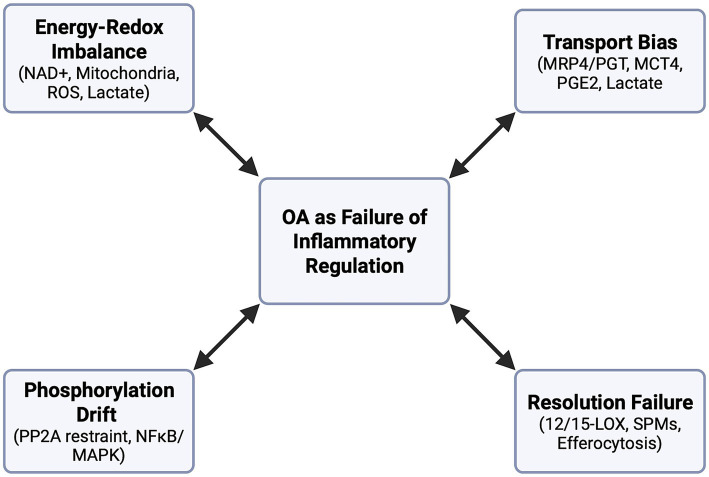
Proposed systems-level model of OA as failure of inflammatory regulation. The schematic illustrates four interacting regulatory axes rather than a mandatory linear sequence. Each axis generates testable predictions regarding synovial metabolism, transport, kinase-phosphatase balance, and resolution capacity.

Clinically, the framework is most immediately relevant to biomarker development, patient stratification, and preclinical therapeutic prioritization rather than direct treatment selection. Patients with prominent synovitis, metabolic disease, post-traumatic OA, or pain disproportionate to radiographic change may represent distinct regulatory phenotypes. Future studies should test whether these phenotypes differ in redox state, transporter bias, PP2A activity, and resolution capacity, and whether such differences predict response to targeted interventions (Figure 1).

## 7 Conclusion

This Hypothesis and Theory article proposes that OA can be studied as a failure of inflammatory regulation and resolution rather than only as the terminal consequence of cartilage wear. The model integrates aging-related redox decline, mediator transport, kinase-phosphatase imbalance, and defective resolution into a falsifiable framework. Its principal value is not that it establishes a new consensus, but that it identifies measurable control points whose validation could improve biomarker development, patient stratification, and future disease-modifying strategies.

## Data Availability

The original contributions presented in the study are included in the article/supplementary material, further inquiries can be directed to the corresponding author.
